# Physical activity promotion practice within primary care: a cross-sectional survey of primary care health professionals in England

**DOI:** 10.1136/bmjopen-2024-093632

**Published:** 2025-07-13

**Authors:** Jimi Osinaike, Robert J Copeland, Anna Myers, Sarah J Hardcastle

**Affiliations:** 1 School of Sport and Physical Activity, Sheffield Hallam University, Sheffield, UK; 2 Advanced Wellbeing Research Centre, Sheffield, Sheffield Hallam University, Sheffield, UK

**Keywords:** Primary Health Care, PUBLIC HEALTH, Primary Prevention, Primary Care

## Abstract

**Abstract:**

**Objectives:**

To investigate physical activity (PA) promotion practices among primary care health professionals in England. To assess whether attitudes, confidence, role perceptions, knowledge of PA guidelines, and PA behaviour were related to PA promotion practices. To examine the barriers to and facilitators of PA promotion practices.

**Design:**

A cross-sectional online survey study with open (free text) questions.

**Setting:**

National survey and online-administered survey conducted in England.

**Outcome measures:**

The outcome variables were attitudes, confidence, role perceptions, PA behaviour, knowledge of the PA guidelines and PA promotion practices. Structural equation modelling evaluated associations between these variables.

**Participants:**

A total of 181 primary care healthcare professionals completed an online survey. The majority were general practitioners (GPs) (66.7%), followed by first contact physiotherapists (13.8%), practice nurses (12.2%) and link workers (7.7%).

**Results:**

Most (59%) healthcare professionals did not meet recommended levels of PA and could not accurately identify the PA guidelines (53%). Most provided PA advice to patients but fewer than 40% assessed PA, supported behaviour change or made referrals to PA support programmes. More first contact physiotherapists and link workers reported more frequent engagement in collaborative aspects of PA promotion, including assessing PA motivation, supporting behaviour change and providing follow-up. Confidence in promoting PA (β=0.30, p<0.001) and positive attitudes (β=0.30, p<0.001) were the only significant predictors of PA promotion practices. Positive associations were observed between confidence, attitudes, PA behaviour and PA promotion practices. Barriers to PA promotion included time constraints and limited and affordable local PA programmes. Facilitators included time and affordable local PA programmes.

**Conclusions:**

Most primary care professionals routinely provide PA advice and feel confident doing so. However, with fewer than half able to accurately recall current PA guidelines and routine assessment and behaviour change support rarely reported, the quality and specificity of this advice remain unclear. While time constraints remain a major barrier to PA promotion, particularly among GPs, the addition of first contact physiotherapists and link workers is likely to enhance capacity for promoting PA in busy primary care settings.

**Strengths and limitations of this study**

- This study included the perspectives of first contact physiotherapists and link workers who are recent additions to the primary care workforce in England.
- This study used structural equation modelling, a multivariate analysis approach, to explore the relationship between physical activity (PA) knowledge, behaviour, attitudes, confidence and PA promotion practices, enhancing the depth of analysis.
- The cross-sectional nature of the study precludes the ability to determine causality between variables.
- The relatively small sample size and convenience sampling may have affected the representation and generalisability of findings.
- Social desirability may have influenced responses, leading to an overestimation of PA behaviour, confidence and PA promotion practices.

## Introduction

 Evidence supports the pivotal role of physical activity (PA) in preventing and managing non-communicable disease (NCD).^1^ PA guidelines in the UK recommend that adults participate in at least 150 min of moderate-intensity aerobic exercise per week, and 2 weekly sessions targeting muscle strength, to attain clinically meaningful health benefits.^2^ More than one-third of adults in the UK do not meet these recommendations, with participation following a social gradient.^3^ On average, adults in the UK visit their general practitioners (GPs) approximately five times a year, and half of these consultations relate to managing long-term health conditions.^4^ Primary care settings, therefore, offer an opportune platform to engage a large portion of the population in health promotion efforts.^5^ Moreover, GPs are perceived as trusted sources of health information and lifestyle advice^6^ and regularly encounter patients who could benefit from increased PA to prevent or manage long-term health conditions.^6^ Primary care settings and GPs are ideally positioned to promote PA to patients. Despite the potential reach of GPs, it has been noted that many do not discuss PA and access their patients PA levels.^7^ With an average of three medical issues addressed per patient visit,^8^ time emerges as the most significant barrier to PA promotion. Notably, GPs' PA behaviour and awareness of local PA opportunities have been cited as key facilitators of PA promotion practices.^9^ In the UK, findings among GPs show that despite positive attitudes towards PA and confidence to raise the topic of PA with patients, many were not familiar with the national PA guidelines.^9^,^11^ The quality and appropriateness of PA advice given to patients and whether this aligns with the PA guidelines is unclear.

Recent policy changes in the UK have created an enhanced opportunity for involving multiple health professionals in promoting PA.^12^ Primary care networks (PCNs) have been set up, introducing new roles including link workers and first contact physiotherapists as front-line primary care practitioners.^13 14^ Link workers, through social prescribing, provide a means for PCNs to link individuals with local PA opportunities. A qualitative investigation into the effects of a link worker social prescribing initiative revealed that social prescribing resulted in beneficial physical and behavioural transformations, such as weight reduction and increased PA.^15^ These changes in the UK primary care system unfolded amidst the backdrop of the COVID-19 pandemic, which imposed unparalleled pressures on primary care.^16^ The pandemic exacerbated pre-existing health disparities, with disadvantaged communities bearing a disproportionate burden.^17^ Moreover, population-wide declines in PA were observed, associated with periods of national lockdown, impacting specific groups disproportionately, including the elderly, individuals of black, Asian, or minority ethnicity, and those with underlying health conditions.^18^ The growth in social prescribing networks and the introduction of link workers and first contact physiotherapists might provide enhanced capacity for much-needed PA promotion in primary care and create opportunities to also address known inequalities in PA participation.

Considering that time constraints limit the ability of GPs to promote PA,^9^ there have been calls for other primary care health professionals to get involved in PA promotion.^19^ The integration of link workers and first contact physiotherapists into primary care pathways offers a potential avenue to enhance the capacity for PA promotion. These roles may support more routine PA assessment and contribute to the dissemination of PA guidelines across patient populations, which is not routine practice. However, there remains a gap in knowledge concerning the attitudes, confidence and PA promotion practices of link workers and first contact physiotherapists. Addressing this gap is essential for understanding the extent to which these allied health professionals can contribute to PA promotion efforts within primary care. Furthermore, the evolving composition of the primary care workforce and changes in work practices since the COVID-19 pandemic, with many consultations being offered remotely rather than face-to-face, highlight the value of an update concerning PA promotion practices. Therefore, the primary aim of this study was to investigate PA promotion practices among health professionals working in primary care in England and the relationship between attitudes, confidence, role perceptions, knowledge of PA guidelines, PA behaviour and PA promotion practices. A secondary aim was to examine the barriers to and facilitators of PA promotion practices.

## Methods

### Study design

This study employed a cross-sectional survey design. An online questionnaire was used to engage primary care health professionals from across England. The survey was designed and hosted using the Qualtrics XM software.^20^ A deliberate convenient non-probability sample of the population of primary care health professionals in England was recruited. Though, a convenient non-probability sample lacks a predetermined sample size, it has been shown to be methodologically acceptable when priority is accessibility and feasibility over-representativeness, especially in research settings where the target population is difficult to access, such as healthcare professionals.^21^ Furthermore, this sampling strategy was deemed appropriate, given that the focus was on generating hypotheses rather than on the generalisability of findings.

### Procedures

The online survey was distributed between June 2023 and August 2023 to GPs, practice nurses, link workers and first contact physiotherapists currently practising in England. The local medical committee of the National Health Service and the Association of Primary Care Managers agreed to distribute the questionnaire to their members via their internal mailing system, and through an advert on their monthly news bulletin. Furthermore, the questionnaire was also shared on X (formerly Twitter) and LinkedIn. Snowball sampling was also employed to reach broader circles of primary care health professionals. As part of the online survey, participants were presented with a brief introductory statement, which included participant information and informed consent. Once written consent was provided, participants were able to complete the survey anonymously.

#### Study instrument

Survey items were developed following a literature review on PA promotion in primary care. Items assessing knowledge of PA guidelines, as well as attitudes and behaviours related to PA promotion, were adapted from studies examining the knowledge, attitude and PA promotion practices among primary care providers and oncologists.^11 22^ Items assessing confidence in giving PA advice were drawn from a study on GPs’ PA promotion practices.^9^ Items on roles and practices were adapted from a review of primary care behavioural counselling interventions,^23^ while those on barriers were based on a systematic review of PA promotion delivery in primary care.^24^ The survey had eight sections (online supplemental file 1) including (1) participant demographics, including sex, age, role, years in practice, and location of practice; (2) knowledge of the PA guidelines (two items, one for aerobic guideline and one for the muscle strengthening guidelines); (3) PA behaviour (single item); (4) confidence to provide general advice on PA to patients (single item); (5) attitudes towards PA for various health conditions using six items, which showed a high internal consistency (Cronbach’s alpha, α=0.92); (6) perceived PA promotion role using five items and this demonstrated a high internal consistency (Cronbach’s alpha, α=0.74); (7) PA promotion practices using six items, and this displayed a high internal consistency (Cronbach’s alpha, α=0.91) and (8) PA promotion barriers using five items (displayed low internal consistency; Cronbach’s alpha, α=0.53). In addition, to assess the clarity of the questions, pilot testing of the survey was conducted among GPs (n=3), practice nurses (n=2), first contact physiotherapists (n=1) and link workers (n=1). Feedback from the pilot testing indicated that the survey was generally well understood, with only minor syntactic modifications required to improve clarity and flow. For example, a question on PA promotion practice originally phrased as *“I signpost patients to physical activity services”* was revised to “*I refer patients to a local exercise/PA programme?”* to ensure broader understanding.

### Data analysis

Data analysis was performed using IBM SPSS V.26.0 (IBM)^25^ and AMOS V.21.0 (IBM)^26^ software. Statistical significance was set at p=0.05. Categorical data and the sociodemographic information were analysed using descriptive statistics. Knowledge of the guidelines was coded as ‘yes’ or ‘no’ based on the recommended PA guidelines (ie, 150 min of moderate-intensity PA per week and twice a week of strength training). The open-ended responses were analysed using content analysis and inductive coding. Content analysis was chosen because it allows for analysis of text-based data, either written transcripts of verbal interactions or documents created in written form.^27^ A coding frame was devised inductively from the data, with similar codes collated to form themes. The first author (JO) conducted the initial coding by carefully reading and re-reading the responses, generating preliminary codes grounded in the data. These codes were then grouped into broader categories, from which initial themes were developed. To broaden interpretations and establish credibility, a second author (SH), with a wealth of qualitative researcher experience, reviewed the codes, themes and offered feedback and suggested refinements. This collaborative and iterative process strengthened the analysis and supported the development of coherent themes grounded in the data.

**Figure 1:**
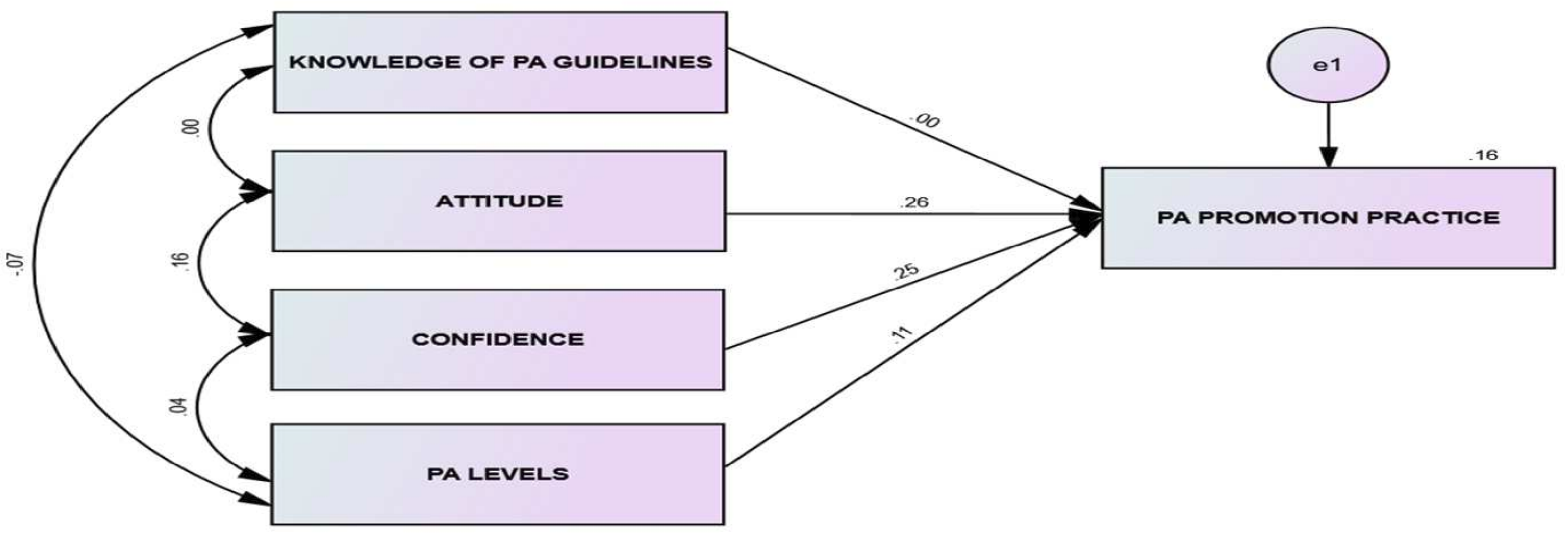
Hypothetical model of variables related to PA promotion. Standardised pathway estimates and multiple correlation (r^2^) associated with PA promotion practices mediated through knowledge of PA guidelines, PA attitudes and confidence to provide PA advice and PA levels. E1, Error term; PA, physical activity.

Pearson’s correlation coefficients were used to analyse the linear relationships between variables. Structural equation modelling (SEM) was used to explore interrelationships among variables for model testing. SEM provides researchers with a flexible framework for developing and analysing complex relationships among multiple variables while testing the validity of theory using empirical models.^28^ SEM was employed to test the fit of the hypothetical model (figure 1). The hypothetical model included four predictor observed variables: PA knowledge, PA attitude, PA levels and confidence to provide PA advice as predictors of the outcome variable (PA promotion).^22^ The path analysis model estimated approximately 15 parameters (see figure 1), namely four regression paths (ie, from each predictor → PA Promotion), four variances (ie, one for each predictor), six covariances among predictors and one error variance for the outcome variable. With a sample size of 181 participants and 15 parameters, the study exceeds the commonly recommended 10:1 participant-to-parameter ratio^29^ and meets established guidelines for sample adequacy in path analysis using observed variables.^29^ Therefore, the sample size is considered sufficient to yield stable and interpretable model estimates. In addition, we made use of the maximum likelihood (ML) method, assuming multidimensional normal distribution to test the fit of the hypothetical model (figure 1). ML is an iterative process that maximises the likelihood that the observed data are most probable or were drawn from its population.^30^ ML often requires a large sample size. Due to the small sample size of this study and the need to accommodate possible non-normality of data, a bootstrapping procedure based on 1000 draws was used.^31^

The overall SEM fit was evaluated using the standardised root mean square residual (SRMR) and the comparative fit index (CFI). The SRMR measures the difference between the observed correlation and the model predicted observation, with lower than 0.08 indicating an acceptable fit.^32^ The CFI measures the extent to which the model of interest is better than an alternative model where measured variables are uncorrelated; values closer to 1 are considered acceptable fit. For this study, CFI values ≥0.9 were considered indicative of good model fit.^33^ The root mean square error of approximation and χ^2^ test were not reported for this study as it has been shown to underestimate model fit with small sample sizes.^34^

### Patient and public involvement

No patients or the public were involved in this research.

## Results

Table 1 provides an overview of participant characteristics. A total of 181 primary care health professionals practising across eight primary care regions in England participated in this study. Most participants were female (60.2%) and aged between 26 and 45 (60.2%). Regarding clinical specialisation, most were GPs (66.7%), followed by first contact physiotherapists (13.8%), practice nurses (12.2%) and link workers (7.7%).

**Table 1:** Participants characteristics

Characteristics	n (%)
Professional role	
GP	120 (66.7)
PN	22 (12.2)
FCP	25 (13.8)
LW	14 (7.7)
Age	
18–25	1 (0.5)
26–45	109 (60.2)
46–55	54 (29.8)
56–65	17 (9.4)
Gender	
Male	71 (38.7)
Female	109 (60.2)
Others	1 (0.5)
Duration of practice	
<6 months	16 (8.8)
7–12 months	19 (10.5)
1–5 years	52 (28.7)
6–10 years	21 (11.6)
>10 years	73 (40.3)
Primary care practice location (region)	
Northwest	72 (39.8)
Yorkshire and the Humber	31 (17.1)
North east	25 (13.8)
South east	23 (12.7)
West Midlands	17 (9.4)
East Midlands	6 (3.3)
East of England	5 (2.8)
South west	2 (1.1)

FCP, first contact physiotherapist; GP, general practitioner; LW, link worker; PN, practice nurse.

Table 2 provides an overview of the self-reported knowledge of PA guidelines, PA behaviour practice and confidence to provide PA advice. Less than half (47.5%) correctly identified the aerobic PA guidelines, and only 14.4% reported the correct strength training guidelines. Among all participants, a larger number of GPs (51.7%) and link workers (21.4%) correctly answered the aerobic activity and strength training guidelines respectively. Less than half (40.9%) reported being sufficiently physically active (ie, doing 30 min or more of PA per week on everyday/most days). Most participants (84.5%) reported being moderately or very confident in providing general PA advice. Overall, attitudes towards PA were very positive, with most (§amp;gt;90%) agreeing that PA is beneficial in the prevention and management of NCDs (online supplemental file 2).

**Table 2:** Self-reported knowledge of PA guidelines, PA behaviour practice and confidence to provide PA advice

	Number of respondents who provided the correct answer, n (%)				
	All participantsn (%)	GPn (%)	PNn (%)	FCPn (%)	LWn (%)
How many minutes per week of moderate intensity PA should an adult undertake to meet the current UK physical activity guidelines?	86 (47.5)	62 (51.7)	7 (31.8)	12 (48.0)	5 (35.7)
On how many days per week is it recommended that adults undertake strength training to meet the current UK PA guidelines?	26 (14.4)	16 (13.3)	2 (9.1)	5 (20)	3 (21.4)
In the past 2 weeks, on how many days per week have you done a total of 30 min or more of physical activity, which was enough to raise your breathing rate?					
Everyday/most days	74 (41.0)	50 (42.0)	9 (41.0)	10 (40.0)	5 (36.0)
On about half the day	33 (18.2)	21 (17.5)	3 (13.6)	7 (28.0)	2 (14.3)
A few times/almost never	74 (41.0)	49 (41.0)	10 (45.5)	8 (32.0)	7 (50.0)
How confident are you in giving PA advice					
Very confident/moderately confident	153 (84.5)	97 (80.8)	20 (91.0)	24 (96.0)	12 (86.0)
Neither confident nor unconfident	16 (8.8)	14 (11.7)	1 (4.6)	0	1 (7.1)
Slightly unconfident/not at all confident	12 (6.6)	9 (7.5)	1 (4.6)	1 (4.0)	2 (14.3)

FCP, first contact physiotherapist; GP, general practitioner; LW, link workers; PA, physical activity; PN, practice nurse.

Table 3 provides an overview of the PA promotion roles and practices. More than 50% of participants agreed or strongly agreed that their PA promotion role included assessment of patient PA, motivating patients to be active, offering PA recommendations and assisting with behaviour change. Fewer than 50% agreed or strongly agreed that referring patients to PA programmes was part of their role. Among professional groups, more first contact physiotherapists agreed or strongly agreed that all PA promotion components (assessing patients’ PA levels, motivating them to be active, offering PA recommendations, assisting with behaviour change and referring patients to PA programmes) were part of their role.

**Table 3:** PA role perception and PA promotion practices

	All respondentsn (%)	GPn (%)	PNn (%)	FCPn (%)	LWn (%)
PA perceived roles					
It is my role to assess patients’ PA level					
Strongly agree/agree	106 (58.6)	70 (58.3)	9 (40.1)	21 (84.0)	6 (43.0)
Neither agree nor disagree	48 (26.5)	33 (27.5)	7 (32.0)	3 (12.0)	5 (36.0)
Disagree/strongly disagree	27 (15.0)	17 (14.2)	6 (27.3)	1 (4.0)	3 (21.4)
It is my role to provide specific PA recommendations to patients					
Strongly agree/agree	106 (56.0)	66 (55.0)	11 (50.0)	23 (92.0)	6 (7.0)
Neither agree nor disagree	46 (25.4)	33 (28.0)	6 (27.3)	2 (8.0)	5 (42.9)
Disagree/strongly disagree	29 (16.0)	21 (18.0)	5 (23.0)	0	3 (21.4)
It is my role to assess motivation to become physically active					
Strongly agree/agree	118 (65.2)	73 (60.8)	15 (68.2)	24 (96.0)	6 (42.9)
Neither agree nor disagree	43 (23.8)	31 (26.0)	4 (18.2)	1 (4.0)	7 (50.0)
Disagree/strongly disagree	20 (11.1)	17 (14.2)	3 (13.6)	0	1 (7.1)
It is my role to assist patients with PA behaviour change					
Strongly agree/agree	119 (65.7)	79 (65.8)	11 (50.0)	23 (92.0)	6 (42.9)
Neither agree nor disagree	35 (19.3)	25 (20.8)	3 (14.0)	1 (4.0)	6 (42.9)
Disagree/strongly disagree	27 (14.9)	16 (13.3)	8 (36.4)	1 (4.0)	2 (14.3)
It is my role to arrange for follow-up and re-evaluate patient’s PA behaviour change					
Strongly agree/agree	63 (34.8)	38 (30.0)	8 (36.4)	12 (48.0)	5 (35.7)
Neither agree nor disagree	53 (29.3)	37 (30.8)	3 (14.0)	6 (24.0)	7 (50.0)
Disagree/strongly disagree	65 (36.0)	45 (31.6)	11 (50.0)	7 (28.0)	2 (14.3)
PA promotion practices					
I give patients PA advice					
Everyday/most days	110 (60.3)	64 (53.3)	14 (63.6)	24 (96.0)	8 (57.1)
On about half the days	24 (13.3)	20 (16.7)	3 (13.6)	0	1 (7.1)
A few times/almost never	47 (23.2)	36 (30.0)	5 (22.7)	1 (4.0)	5 (35.7)
I assess patient PA level					
Everyday/most days	73 (40.3)	43 (36.0)	6 (27.3)	20 (80.0)	4 (28.6)
On about half the days	42 (23.2)	36 (21.7)	5 (22.7)	4 (16.0)	7 (50.0)
A few times/almost never	66 (36.5)	51 (43.0)	11 (50.0)	1 (4.0)	3 (21.4)
I help patient with PA behaviour change					
Everyday/most days	81 (44.8)	43 (35.8)	12 (54.5)	20 (80.0)	6 (42.9)
On about half the days	34 (18.8)	24 (20.0)	2 (9.1)	4 (16.0)	4 (28.6)
A few times/almost never	66 (36.5)	53 (44.2)	8 (36.4)	1 (4.0)	4 (28.6)
I assess patients’ motivation to become physically active					
Everyday/most days	74 (40.9)	40 (33.3)	7 (31.8)	10 (80.0)	7 (50.0)
On about half the days	38 (21.0)	32 (26.7)	1 (4.6)	2 (8.0)	3 (21.4)
A few times/almost never	69 (38.1)	48 (40.0)	14 (63.6)	3 (12.0)	4 (28.6)
I follow-up with PA behaviour change					
Everyday/most days	42 (23.2)	21 (18.0)	6 (27.3)	9 (36.0)	6 (42.9)
On about half the days	12 (6.6)	8 (6.7)	1 (4.6)	2 (8.0)	1 (7.1)
A few times/almost never	127 (70.2)	91 (76.0)	15 (68.2)	14 (56.0)	7 (50.0)
I refer patients to a local exercise/PA programme?					
Everyday/most days	67 (37.0)	45 (37.5)	7 (31.9)	10 (40.0)	5 (35.7)
On about half the days	34 (18.8)	20 (16.7)	4 (18.2)	7 (28.0)	3 (21.4)
A few times/almost never	80 (44.2)	55 (45.8)	11 (50.0)	8 (32.0)	6 (42.9)

FCP, first contact physiotherapist; GP, general practitioner; LW, link workers; PA, physical activity; PN, practice nurse.

Most participants (60.3%) reported routinely (everyday/most days) providing PA advice (table 3). Less than 40% of participants routinely assessed PA behaviour, assisted with PA behaviour change, evaluated patient motivation for PA, followed up on PA behaviour change, or referred patients to local exercise programmes. Among professional groups, more first contact physiotherapists reported routinely (everyday/most days) engaging in all aspects of PA promotion, except for following up on PA behaviour change.

Among the predetermined barriers to PA promotion, time constraints were identified as the main barrier to PA promotion by 51.4% of all participants (online supplemental file 3). Most GPs (62.5%) agreed that a lack of time was a barrier, compared with 49.9% of practice nurses, 13% of first contact physiotherapists and 28.6% of link workers. Over one-third (36%) agreed or strongly agreed that patients are unlikely to follow their advice to be physically active. Similarly, 39% of participants agreed or strongly agreed that there are no local PA programmes to refer patients to.

### Structural equation modelling

The hypothetical model included PA knowledge, attitude, PA behaviour, PA promotion role and confidence to provide PA advice as observed variables predicting PA promotion, which was the outcome variable. This model did not show a good fit (CFI=0.69; SRMR=0.09). Standardised parameter estimates indicated that only attitude towards PA (β=0.22, p<0.001) and confidence in promoting PA (β=0.30, p <0.001) significantly predicted PA promotion practices. PA role perception (β=−0.14, p=0.04) reported a negative significant pathway in predicting PA promotion. PA behaviour (β=0.10, p=0.22) and knowledge of the PA guidelines (β= 0.00, p=0.91) reported a non-significant pathway to predicting PA promotion practices.

To improve the model fit, a recommended procedure is to remove some variables and or include additional paths, or inclusion of loops (covariance) that correlates the observed variables together.^29^ Thus, to improve on the model fit, PA role perception was removed due to its negative predictive pathway. In addition, inclusion loops were then added to correlate attitudes towards PA and confidence to promote PA. Figure 1 shows the new hypothetical model after adjustment. This new model reported a good model fit (CFI=1.00; SRMR=0.04). Standardised parameter estimates indicate that confidence to promote PA (β=0.30, p<0.001) and attitude towards PA (β=0.30, p<0.001) reported a significant direct positive pathway to predict PA promotion practices.

### Correlation analysis

PA promotion practices were significantly positively correlated with confidence to promote PA (r=0.34, p<0.001), attitudes towards PA (r=0.31, p<0.001) and PA levels (r=0.15, p<0.001). Confidence to promote PA was positively correlated with attitude towards PA (r=0.20, p<0.001) (online supplemental file 4).

### Open-text responses of barriers and facilitators to PA promotion

The open-text questions promotion yielded 112 responses identifying barriers and 120 responses regarding facilitators for PA promotion (table 4). The predominant barriers reported were time constraints (n=50, 44.6%), limited availability and affordability of local PA opportunities (n=27, 24.1%) and perceived patient disinterest in PA (n=24, 21.4%). Other barriers included insufficient PA referrals and knowledge of local opportunities (n=4, 4%), a lack of training in PA counselling (n=3, 3%), confidence to promote PA (n=2, 2%) and access to clear referral pathways/systems (n=2, 2%). Besides having more time in consultations, which was the most cited facilitator of PA promotion (n=46, 38.3%), other facilitators include access to affordable local PA opportunities (n=27, 22.5%), engagement of other healthcare professionals in PA promotion (n=22, 18.3%) and access to PA promotion resources (n=15, 12.5%) (see table 4 for themes and corresponding quotes).

**Table 4:** Open-text responses to perceived barriers to and facilitators of PA promotion

Themes: What would most help you to regularly discuss and promote PA to your patients	Frequency, n (%)	Example responses
More time	46 (38.3)	“Having longer appointment. times to explore behaviour change.” FCP“Having allocated protected time.” GP
Access to affordable local PA opportunities	27 (22.5)	“Having more services to signpost people to that are affordable.” LW“Having good quality places that are affordable that we could refer people to.” PN
Engagement of other healthcare professionals in PA promotion	22 (18.3)	“Nurses probably better placed and do so in for example, diabetes/hypertension reviews.” GP“Access to a colleague who can give specific advice-have health coaches but also think first contact physio would be useful.” PN
Access to PA promotion resources	15 (12.5)	“Perhaps some information in the waiting areas that this may be discussed in your consultation”. GP“Leaflets/Posters and easier during nurse’s appointments” PN
Need for wider systems change	6 (5)	“General public being encouraged to exercise so we are all giving the same advice.” GP“Schools and workplace changes to support more exercise within daily life.” GP.
Patients understanding of the importance of PA	4 (3.3)	“patient’s awareness and understanding on this.” GP“If patients demonstrated adherence to even basic exercises.” FCP

Themes: What other barriers to physical activity promotion affects your current physical activity promotion practices?	Frequency, n (%)	Example responses
Time constraints	50 (44.6)	“The main issue is within the consultation particularly with the increasing complexity of conditions presenting to GP.” GP“Limited in a 10 min consultation to do the issues they arrived for let alone the physical exercise advise.” PN
Limited availability and affordability of local PA opportunities	27 (24.1)	“Limited physical activity program in community to refer to.” FCP“There are no free or affordable local PA opportunities to refer patients to.” LW
Perceived patient disinterest in PA advice and referral	24 (21.4)	“Patients tend to not want referral to the exercise programme. Also mostly are not interested in the advice.” GP“Patients often have other priorities for their 10 minutes.” GP
Insufficient PA referrals and knowledge of local opportunities	4 (4.0)	“Not knowing what is offered.” LW“As a link worker I hardly ever get referrals related to improving patient physical activity.” LW
Lack of PA counselling training	3 (3.0)	“As nurse we lack the ability for continuous coaching to keep patients on track” PN“I would usually refer to health coaches as they have more training then I do.” LW
Confidence to promote PA	2 (2.0)	“Patients have so many barriers to physical activity that I sometimes avoid promoting it as they are more concerned about their depression…. etc.”““Sometimes difficulty to engage in a conversation about activity if someone comes in with an issue that does not relate to activity for example, thumb OA.” FCP
Access to clear referral pathways/systems	2 (2)	“…. sometimes confusing referral pathways.” GP“……not part of NHS pathways/systems” GP

PA, physical activity.

## Discussion

This study found that while most primary care health professionals reported confidently giving PA advice, they rarely reported engaging in PA promotion tasks such as assessing patient’ PA level, assessment of PA behaviour and motivation, and follow-up on behaviour change. However, first contact physiotherapists and link workers reported promoting these PA components more frequently, suggesting an enhanced capacity for promoting PA within busy primary care settings. Limited availability and affordability of local PA opportunities was often cited as a barrier to PA promotion, suggesting that primary care health professionals have a mindset of referring patients to supervised, facility-based exercise programmes rather than promoting lifestyle-based activities such as independent outdoor walking.

Consistent with previous research, a high proportion of primary care professionals (60.3%) report regularly providing PA advice to patients and express confidence in doing so.^9 11^ However, they were unable to cite the PA guidelines (ie, aerobic and strength PA guidelines), thereby raising concerns about whether their advice aligns with PA guidelines aligns with the PA guidelines or indeed whether it entails specific advice. While previous literature has predominantly highlighted gaps in PA guideline knowledge among GPs, practice nurses and physiotherapists,^9 11 35 36^ our findings extend this concern to link workers. As primary care grows more multidisciplinary, embedding PA guideline training into induction and ongoing education is essential. The WHO PA guidance recommends at least 150 min of moderate or 75 min of vigorous intensity PA weekly but also emphasises that any movement benefits health.^37^ Clinicians should endorse formal PA guidelines while reinforcing the broader ‘move more’ message. Given that patients often cite time, family commitments and fatigue as barriers,^38^ encouraging any increase in activity is likely to be a pragmatic entry point to becoming physically active. Ultimately, consistent PA promotion depends not only on clinicians’ knowledge and confidence, but also on their perceptions of patients’ ability to meet recommended PA intensities.

Consistent with previous research,^39 40^ most primary care professionals did not routinely assess PA. Routine PA assessment is critical for monitoring inactivity,^30^ offering insights into health status and providing a baseline for PA intervention. Despite the recognised value of PA assessments, an earlier study has highlighted challenges particularly among GPs and practice nurses who cite time constraints and the complexity of consultations as barriers to the use of standardised PA assessment tools like the General Practice Physical Activity Questionnaire.^41^ Notably, 80% of first contact physiotherapists reported routinely assessing PA, possibly reflecting its central role in their practice or greater consultation time. However, it is unclear whether assessments used validated tools or relied on subjective judgement. These findings highlight the potential role of physiotherapists in embedding PA assessment into routine care and underscore the need for further research into assessment methods, the underlying rationale for existing practices, and patients’ perspectives on acceptable PA assessment.

Most primary care professionals report limited involvement in key collaborative aspects of PA promotion such as supporting behaviour change, exploring motivation and follow-up, despite their proven role in sustaining PA improvements.^42^ Our findings align with previous research among GPs^11 40 43 44^ and may reflect structural and perceptual barriers that persist within general practice, including short consultation times, low self-efficacy in behaviour change counselling and assumptions about patient disinterest.^24^ In contrast, first-contact physiotherapists and link workers report greater engagement in behaviour change support, possibly due to longer appointments, focused scope of practice in PA, and potentially greater priority towards PA for health. While their involvement could boost PA promotion capacity, particularly given GPs’ limited time and clinical demands,^9 19 44^ limited access to patients compared with GPs raises concerns about reach and equity. GPs remain uniquely positioned to reach diverse and complex patients and are trusted health advisors.^6^ Therefore, sole reliance on allied health professionals’ risks fragmenting PA promotion and framing it as a ‘specialised’ task rather than a core component of routine primary care. To address this, PA promotion must be reconceptualised as a shared primary care responsibility. System-level interventions such as embedding PA-related quality indicators in performance frameworks^45^ could incentivise PA promotion, while integrating behaviour change competencies and fostering interdisciplinary collaboration may normalise it as standard care. Additionally, leveraging digital tools to support motivational screening, brief interventions and automated follow-ups has demonstrated efficacy in improving patient PA levels and may alleviate the time burden on clinicians while enhancing care continuity.^46^

A novel finding from the present study was an apparent mindset among participants favouring facility-based or supervised PA, as revealed through the qualitative responses (ie, the citing of limited availability and affordability of local PA opportunities as a barrier to PA promotion), potentially narrowing the scope of PA promotion. This suggests primary care professionals tend to prioritise referrals to supervised programmes over lifestyle activities like walking or cycling, which patients reportedly prefer.^47^ Therefore, the promotion of lifestyle-based PA is likely to be beneficial, as it eliminates barriers of cost and travel associated with facility-based PA.^48^ Although link workers are well placed to promote activity through social prescribing, few GPs and practice nurses referred patients to them, perhaps due to limited role awareness or a preference for supervised, facility-based PA referrals. Going forward, GPs should prioritise independent self-managed lifestyle activities such as walking as a key form of PA, supported by referrals to link workers who can connect patients who may need extra motivation to local walking opportunities. As link workers become further embedded in primary care, increasing clinician awareness of their role in facilitating accessible PA will be essential.

Patient disinterest in PA advice was also cited as a barrier to PA promotion, and this has been reported previously among primary care health professionals.^10 24^ However, this perception may not accurately reflect patient attitudes towards PA promotion. Evidence suggests that patients often report receiving little to no PA advice during clinical encounters, and when such advice is provided, it is frequently described as vague, generic or impersonal.^49^,^51^ Notably, patient receptivity to PA advice appears to increase substantially when the communication is individualised and contextualised, particularly when linked to specific health outcomes such as pain reduction or decreased dependence on medication.^49^ This mismatch may stem from communication misperceptions rather than genuine patient disengagement, underscoring the need for personalised PA advice. If clinicians cannot tailor PA guidance due to time, training or confidence constraints, engagement suffers, perpetuating the false belief that patients lack interest. Ultimately, this may suggests a systemic communication issue rather than true patient disinterest. Further research is however needed to explore patient interest in PA promotion and what such advice should entail. Additionally, more investigation with healthcare professionals is required to understand the reasoning behind the perception that patients are disinterested in PA. Gaining insight into healthcare professionals' views could help identify the barriers or misconceptions influencing PA promotion, ensuring that interventions are better aligned with patient preferences and needs.

Consistent with a prior study,^52^ most healthcare professionals (59%) failed to achieve the aerobic PA guidelines. Existing literature suggests an association between the personal PA behaviour of healthcare professionals and their PA promotion practice.^19 53 54^ PA behaviour did not predict PA promotion in the present study despite being positively related. This suggests that personal activity levels alone may be insufficient to drive consistent PA promotion. The absence of a predictive relationship could reflect the influence of mediating factors such as confidence, perceived role adequacy, or systemic barriers such as time constraints to promotion. Importantly, it is somewhat unclear whether physically active healthcare professionals more frequently promote PA, and further work is needed to ascertain this using objective measures of healthcare professional PA behaviour. Overall, these findings suggest that healthcare professionals' PA behaviour may impact PA promotion practices through attitudes and confidence. Improving healthcare professionals’ PA engagement may increase PA promotion.

### Study strengths and limitations

Strengths of the study include the inclusion of the perspectives of first contact physiotherapists and link workers who are recent additions to the primary care workforce in England and the national representation. Additionally, the study used SEM, a multivariate analysis approach,^28^ to explore the relationship between PA knowledge, behaviour, attitudes, confidence and PA promotion practices, enhancing the depth of analysis. Limitations include the cross-sectional nature of the study precluding the ability to determine causality between variables, relatively small sample size, and the use of a convenience sampling strategy may have affected the generalisability of findings.^55^ Social desirability^56^ may have also influenced responses, leading to an overestimation of PA behaviour, confidence and PA promotion practices. Due to the use of snowball sampling and survey distribution via social media and internal mailing system of the professional groups, it was not possible to determine the total number of individuals who received or viewed the survey invitation. As such, the non-response rate could not be determined. Another limitation of this study is the absence of validated questionnaires on this topic. As a result, pragmatic decisions were made regarding the questions used, which were developed from previous research^10 11^ in the field.

### Implications for research and practice

Primary care professionals report confidence in providing PA advice to patients but gaps in their knowledge of PA guidelines, limited PA assessment and behaviour change support raise concerns about the quality of PA advice given and its effectiveness. Research is needed to clarify current PA messaging and its rationale. Targeted training on PA guidelines and studies on clinicians’ perceptions and readiness to implement them are essential. Given the infrequent use of routine PA assessments, training and incorporation of PA assessments into incentive frameworks such as the Quality and Outcomes Framework^45^ could drive more consistent and effective practice. Perceived patient disinterest may stem from delivery of PA advice rather than genuine disengagement, underscoring the need to enhance clinicians’ behaviour change skills. While time constraints may limit GPs’ ability to offer personalised PA support, this study suggests that first contact physiotherapists and link workers can enhance capacity for promoting PA in busy primary care settings. Further research should examine the current practices of first contact physiotherapists and link workers and explore how they can be effectively supported to deliver tailored PA interventions. Additionally, limited qualitative research^24^ on patient receptivity to PA advice within primary care highlights the need for studies exploring how personalised, context-specific communication strategies can enhance patient engagement.

The emphasis on supervised, facility-based programmes over lifestyle-based activities such as outdoor walking warrants further investigation. Moreover, clearer referral pathways to link workers may be necessary for patients with low motivation or complex needs who may benefit from structured support. Future research should incorporate objective measures to better understand the relationship between healthcare professionals’ own PA behaviours and their promotion practices.

## Conclusions

A high proportion of primary care professionals report regularly providing PA advice to patients and express confidence in doing so. However, since less than half can accurately cite the PA guidelines and routine PA assessment and behaviour change support are rarely reported, the quality and specificity of the PA advice remain unclear. While improving knowledge is necessary, it is unlikely to be sufficient on its own. Given the infrequent PA assessment and behaviour change support observed, further research is warranted to fully explore the nature of PA advice provided, the patients who receive it, and the reasoning behind such PA promotion practices. Additionally, findings suggest a prevailing mindset among primary care professionals that favours facility-based or supervised PA programmes, potentially narrowing the scope of PA promotion. Perceived patient disinterest is frequently cited as a barrier, though this may reflect a communication misperception rather than actual disengagement. With the potential for first-contact physiotherapists and link workers to enhance capacity for promoting PA in busy primary care settings, this study calls for stronger integration of these roles within multidisciplinary teams to foster greater patient engagement in PA.

## Supplementary material

10.1136/bmjopen-2024-093632 online supplemental file 1

## Data Availability

Data are available in a public, open access repository. Data are available on reasonable request. All data relevant to the study are included in the article or uploaded as supplementary information.
