# Supplementary material for: Physical activity promotion practice within primary care: a cross-sectional survey of primary care health professionals in England
Source: BMJ Open. 2025 Jul 13;15(7):e093632. doi: 10.1136/bmjopen-2024-093632 (PMC12273183; doi:10.1136/bmjopen-2024-093632)
Supplement: online supplemental file 1 [file bmjopen-15-7-s001.docx]

Supplementary file 1.

**HEALTHCARE PROFESSIONALS’ VIEWS ON PHYSICAL ACTIVITY PROMOTION IN PRIMARY CARE (ER37660039)**

This survey is for General practitioners, practice nurses, link workers, and first contact physiotherapists (FCP) who are currently practicing within primary care in England.  A general practitioner (GP) is a medical doctor who provides medical services within a GP surgery.  A first contact physiotherapist is a physiotherapist who is based in or works for GP surgeries with an expertise in the assessment and management of MSK conditions.  A link worker is a professional who helps patients to improve their health, wellbeing and social welfare by connecting them to community services.  A nurse practitioner is a registered nurse that works in GP surgeries to assess, screen, treat and educate patients, and help doctors give medical care. You are eligible to complete this survey as a GP, FCP, link worker, or practice nurse regardless of your employment status; full-time or part-time. You are also eligible to complete this survey if you are currently practicing within a GP surgery or primary care in England. This survey should take approximately 20 minutes to complete. This survey consists of three sections, most questions require a tick box response to indicate your answer. You can scroll backwards and forwards at any stage of the survey if you wish to review/change a previous response.

You will be notified once you have reached the end of the survey. If you do not progress to the end of the survey and/or submit your responses, no data will be collected. If you have any queries about this research, please contact: [J.Osinaike@shu.ac.uk](mailto:J.Osinaike@shu.ac.uk)

The participant information sheet for this study can be accessed via the link below.

[Participant information sheet](https://shusls.eu.qualtrics.com/CP/File.php?F=F_ewUBlKmKdo4LKqW)

Consent

If you consent to participate in this survey, please acknowledge your agreement to the following statements by selecting ‘yes’ below if you wish to participate. If you do not agree, please select ‘no’ and submit, this will lead you to the end of the survey.

My questions about the study have been answered to my satisfaction and I understand that I may ask further questions at any point. I understand that my personal data will only be collected. I understand that I am free to withdraw from the study within the time limits outlined in the Information Sheet, without giving a reason for my withdrawal or to decline to answer any questions in the study without any consequences to my future treatment by the researcher.

I understand that the withdrawal of data could be done by contacting the researcher through the email address provided and I also understand that I need to provide my ID code to withdraw my data. I understand that information I provide may be subject to review by responsible individuals from the Sheffield Hallam University.

I understand that information I provide will be used in various anonymised outputs, including report, publication, presentation, website and social media. I understand that my personal data, including this consent form, which link me to the research data, will be kept securely in accordance with data protection guidelines, and only be accessible to the immediate research team or responsible persons at the Sheffield Hallam University. I understand any personal contact details collected about me, such as my name and email address, will not be shared beyond the research team.

I agree to take part in this study. YES/NO

Section 1: Participants demographics.

What is your age (years)?

- 18-25
- 26-45
- 46-55
- 56-65
- ≥66

Location of primary care practice

________________________________________________________________

Professional role

- General practitioner
- Practice nurse.
- Link worker.
- First contact physiotherapist

How long have you been working in your primary care role?

- < 6months
- 7-12months
- 1-5 years
- 6-10 years
- >10 years

Gender

- FEMALE
- MALE
- OTHERS

SECTION 2

How many minutes per week of moderate intensity physical activity should an adult undertake to meet the current UK physical activity guidelines? (Moderate-intensity physical activities are activities that raise your heart rate and make you breathe harder than normal (i.e., brisk walking, moderate cycling)

________________________________________________________________

How many days a week should an adult undertake physical activity to improve their muscle strength to meet the current UK physical activity guidelines?

________________________________________________________________

In the past two weeks, on how many days per week have you done a total of 30 minutes or more of physical activity, which was enough to raise your breathing rate?

- MOST DAYS
- ON ABOUT HALF THE DAY
- A FEW TIMES BUT LESS THAN HALF
- A FEW TIMES
- ALMOST NEVER

SECTION 4: This section assesses your level of confidence in providing PA advise.

|  | Not at all confident | slightly unconfident | Neither confident nor unconfident | Moderately confident | Very confident |
| --- | --- | --- | --- | --- | --- |
| giving general advice to patients on physical activity? |  |  |  |  |  |

SECTION 5: This section will ask about your beliefs regarding the benefits of physical activity for a range of health outcomes.

|  | strongly agree | Agree | Neither agree nor disagree | Disagree | Strongly disagree |
| --- | --- | --- | --- | --- | --- |
| Physical activity reduces the risk of cardiovascular disease |  |  |  |  |  |
| Physical activity is an effective treatment for depression |  |  |  |  |  |
| Physical activity can help treat type 2 diabetes |  |  |  |  |  |
| Physical activity improves mobility and balance |  |  |  |  |  |
| Physical activity both prevent and treat lower back pain |  |  |  |  |  |
| Physical activity prevents the development of osteoarthritis |  |  |  |  |  |

SECTION 6: The purpose of this section is to understand your perceived role as it relates to the promotion of physical activity within your practice.

|  | strongly agree | Agree | Neither agree nor disagree | disagree | strongly disagree |
| --- | --- | --- | --- | --- | --- |
| It is my role to assess patients’ physical activity level |  |  |  |  |  |
| it is my role to provide specific PA recommendations to patients |  |  |  |  |  |
| It is my role to assess patient's willingness to become physically active |  |  |  |  |  |
| It is my role to assist patients with physical activity behaviour change |  |  |  |  |  |
| It is my role to arrange for follow up and re-evaluate patient's physical activity behaviour |  |  |  |  |  |

SECTION 7: The following section will assess your current practice as it relates to how you promote physical activity within your practice. In the past four weeks

|  | everyday | most days | On about half the days | A few times, but less than half | a few times | almost never |
| --- | --- | --- | --- | --- | --- | --- |
| I give patients physical activity advice |  |  |  |  |  |  |
| I assess patient physical activity level |  |  |  |  |  |  |
| I help patient with physical activity behaviour change |  |  |  |  |  |  |
| I assess patient motivation to become physically active |  |  |  |  |  |  |
| I follow-up with physical activity behaviour change |  |  |  |  |  |  |
| I refer patients to a local exercise/PA programme.  . |  |  |  |  |  |  |

SECTION 8: To what extent do the following factors impede your physical activity promotion practices.

|  | strongly agree | agree | neither agree nor disagree | somewhat disagree | disagree |
| --- | --- | --- | --- | --- | --- |
| Patients are unlikely to follow my advice to be more active. |  |  |  |  |  |
| I don’t have the time |  |  |  |  |  |
| There is no financial incentive. |  |  |  |  |  |
| I don’t know how to counsel patients for physical activity |  |  |  |  |  |
| There are no local exercise programmes to refer patients to |  |  |  |  |  |

SECTION 9

What other barriers to physical activity promotion affects your current physical activity promotion practices?

________________________________________________________________

What would most help you to regularly discuss and promote physical activity to your patients?

________________________________________________________________

Thank you for completing this Survey. You will have the opportunity to have access to the findings of this research through your professional organization.

**Supplementary file 2: Attitudes toward PA**

| Benefits of PA | All respondents  n (%) | GP  n (%) | PN  n (%) | FCP  n (%) | LW  n (%) |
| --- | --- | --- | --- | --- | --- |
| PA reduces the risk of cardiovascular disease. |  |  |  |  |  |
| Strongly agree/agree.  Neither agree nor disagree  Disagree/strongly disagree | 176 (97.1)  2 (1.1)  3 (1.6) | 119 (99.0)  1 (1)  0 | 22 (100.0)  0  0 | 21 (84.0)  1 (4.0)  3 (12.0) | 12 (100.0)  0  0 |
| PA is an effective treatment for depression |  |  |  |  |  |
| Strongly agree/agree  Neither agree nor disagree  Disagree/strongly disagree | 168 (93.0)  5 (2.8)  8 (4.4) | 112 (93.3)  8 (6.7)  0 | 21 (95.5)  1 (4.6)  0 | 22 (88.0)  1 (4.0)  2 (8.0) | 14 (100.0)  0  0 |
| PA can help treat type 2 diabetes |  |  |  |  |  |
| Strongly agree/agree  Neither agree nor disagree  Disagree/strongly disagree | 168 (93.0)  8 (4.4)  5 (2.8) | 112 (93.3)  6 (5.0)  2 (1.7) | 21 (95.5)  1 (4.5)  0 | 21 (84.0)  1 (4.0)  3 (12.0) | 14 (100.0)  0  0 |
| PA improves mobility and balance |  |  |  |  |  |
| Strongly agree/agree  Neither agree nor disagree  Disagree/strongly disagree | 174 (96.0)  2 (1.1)  5 (2.8) | 118 (98.0)  1 (1.0)  1 (1.0) | 22 (100.0)  0  0 | 22 (88.0)  0  3 (12.0) | 12 (86.0)  1 (7.0)  1 (7.0) |
| PA both prevent and treat lower back pain |  |  |  |  |  |
| Strongly agree/agree.  Neither agree nor disagree  Disagree/strongly disagree | 174 (96.0)  6 (3.3)  1 (0.6) | 116 (96.7)  3 (2.5)  1 (0.8) | 20 (91.0)  2 (9.1)  0 | 25 (100.0)  0  0 | 13 (93.0)  1 (7.0)  0 |

**Supplementary file 3: Barriers to PA promotion**

|  | All respondents  n (%) | GP  n (%) | PN  n (%) | FCP  n (%) | LW  n (%) |
| --- | --- | --- | --- | --- | --- |
| Patients are unlikely to follow my advice to be more active. |  |  |  |  |  |
| Strongly agree/agree  Neither agree nor disagree  Somewhat disagree/disagree | 65 (35.9)  55 (30.4)  61 (33.7) | 42 (35)  38 (31.7)  40 (33.3) | 10 (45.5)  7 (31.8)  5 (13.6) | 10 (40.0)  9 (36.0)  6 (24.0) | 3 (21.4)  1 (7.1)  10 (71.4) |
| I do not have the time |  |  |  |  |  |
| Strongly agree/agree  Neither agree nor disagree  Somewhat disagree/disagree | 93 (51.4)  28 (15.5)  60 (33.1) | 75 (62.5)  15 (12.5)  30 (25.0) | 11 (49.9)  2 (9.1)  9 (41.0) | 3 (13)  9 (36.3)  13 (59.1) | 4 (28.6)  2 (14.3)  8 (57.1) |
| There are no financial incentives. |  |  |  |  |  |
| Strongly agree/agree  Neither agree nor disagree  Somewhat disagree/disagree | 44 (24.3)  50 (27.6)  87 (48.1) | 32 (26.6)  36 (30.1)  52 (41.7) | 4 (18.1)  6 (27.3)  12 (54.6) | 4 (16)  5 (20.0)  16 (64.0) | 4 (28.6)  3 (21.4)  7 (50.0) |
| I don’t know how to counsel patients for PA |  |  |  |  |  |
| Strongly agree/agree.  Neither agree nor disagree  Somewhat disagree/disagree | 20 (11.1)  29 (16.0)  132 (73.0) | 15 (12.6)  19 (15.8)  86 (72.0) | 3 (13.6)  5 (22.7)  14 (64.0) | 0  5 (20.0)  20 (80.0) | 2 (14.3)  0  12 (86.0) |
| There are no local exercise programmes to refer patients to |  |  |  |  |  |
| Strongly agree/agree.  Neither agree nor disagree  Somewhat disagree/disagree | 39 (21.5)  33 (18.3)  109 (60.2) | 31 (25.9)  2 (19.2)  62 (52.0) | 6 (27.3)  2 (9.1)  17 (77.3) | 2 (8.0)  5 (20.0)  18 (72.0) | 3 (21.4)  3 (21.4)  8 (57.1) |

**Supplementary file 4: Bivariate correlation matrix of PA variables**

|  | Role | Confidence | PA promotion practice | Attitude | Knowledge of PA guideline | PA levels |
| --- | --- | --- | --- | --- | --- | --- |
| Role | 1 |  |  |  |  |  |
| Confidence | -0.07 | 1 |  |  |  |  |
| PA promotion practice | **-0.21**** | **0.34**** | 1 |  |  |  |
| Attitude | **-0.20**** | **0.20**** | **0.31**** | 1 |  |  |
| knowledge of PA guideline | -0.01 | -0.13 | -0.04 | -0.02 | 1 |  |
| PA levels | **-0.18*** | 0.10 | **0.15*** | 0.09 | -0.07 | 1 |

Bold numbers indicate correlations that are statistically significant.

*Correlation is significant at p < .05; **Correlation is significant at p < .01

PA: Physical activity
