# Supplementary material for: Physical activity promotion practice within primary care: a cross-sectional survey of primary care health professionals in England
Source: BMJ Open. 2025 Jul 13;15(7):e093632. doi: 10.1136/bmjopen-2024-093632 (PMC12273183; doi:10.1136/bmjopen-2024-093632)
Supplement: Supplementary file 2 [file bmjopen-2024-093632.reviewer_comments.pdf]

PEER REVIEW HISTORY

BMJ Open publishes all reviews undertaken for accepted manuscripts. Reviewers are asked to complete a checklist review form and are provided with free text boxes to elaborate on their assessment. These free text comments are reproduced below.

ARTICLE DETAILS

Title (Provisional)

PHYSICAL ACTIVITY PROMOTION PRACTICE WITHIN PRIMARY CARE: A SURVEY OF PRIMARY CARE HEALTH PROFESSIONALS IN ENGLAND.

Authors

Osinaike, Jimi; Copeland, Robert; Myers, Anna; Hardcastle, Sarah

---

VERSION 1 - REVIEW

**Reviewer**	**1**
**Name**	**Nordgren, Lena**
**Affiliation**	**Uppsala Universitet, Department of Public Health and Caring Sciences**
**Date**	**09-Dec-2024**
**COI**	**None**

Thank you for the opportunity to review this important and interesting manuscript.

The study addresses a highly relevant and significant topic: the attitudes, knowledge, and behaviors of primary care professionals regarding physical activity and physical activity promotion. This is a timely area of research, particularly given the increasing importance of preventive healthcare and the role of primary care in promoting healthy lifestyles.

One of the study's key strengths is its broad approach, examining various professional groups within primary care, including emerging roles such as first contact physiotherapists and link workers. This provides valuable insights into the diverse perspectives and challenges that exist in promoting physical activity in primary care. Additionally, the use of structural equation modeling as an analytical method is appropriate for exploring the complex relationships between factors such as attitudes, knowledge, and behaviors. However, I recommend reducing the use of abbreviations in both the results and discussion sections. While abbreviations can be helpful, their excessive use can make the text difficult to read and follow, especially when several abbreviations appear in the same sentence.

Specific Comments:

Title:

The title, "Physical Activity Promotion Practice Within Primary Care: A Survey of Primary Care Health Professionals in England", is clear and accurately reflects the scope and content of the study. It effectively communicates the focus on physical activity promotion within primary care and highlights the geographical context, which is valuable for the reader.

Abstract:

The abstract is well-written, accurately summarizing the main objectives, methodology, key results, and conclusions of the study. It provides a clear and concise overview of the research, which effectively engages the reader. The abstract serves its purpose and provides an appropriate summary of the study.

Strengths and Limitations:

I recommend removing all, or at least most, of the abbreviations and using the full terms for clarity. Additionally, I suggest mentioning the sampling strategy (convenience sampling) as a limitation, as it may affect the representativeness and generalizability of the study's findings.

Introduction:

The introduction provides a strong and well-structured background for the study, clearly outlining the significance of physical activity in primary care settings and the challenges healthcare professionals face in promoting it. The rationale for the study is well-justified, particularly in the context of the UK healthcare system and the introduction of new roles such as first contact physiotherapists and link workers. The use of recent policy changes and the impact of COVID-19 on physical activity and healthcare practices further strengthens the relevance and timeliness of the study. Overall, the introduction is clear, well-grounded in existing literature, and effectively sets up the research objectives.

Methods:

• Sample size and nonresponse analysis:

The manuscript does not report on the initial sample size or how many participants were approached but declined to participate. It would be beneficial to include information on the nonresponse rate and consider a nonresponse analysis, where you compare the demographic characteristics of respondents and nonrespondents. This would help assess selection bias and provide insight into how representative the sample is.

• Survey adaptation and outcomes:

While the outcomes of the study are clearly defined and aligned with the research objectives, there is limited information about the process of adapting the survey from previous studies. To enhance transparency, I recommend providing more detail on how the survey was tailored to the context of this study and whether any validation or psychometric evaluation was performed on the adapted questions. Clarifying the adaptation process would strengthen the connection between the survey design and the well-defined outcomes, ensuring that the measures used are valid and reliable.

• Statistical analysis:

The detailed explanation of the statistical methods used, particularly the use of structural equation modeling, bootstrapping, and the fit indices (standardized root mean square residual and comparative fit index), is appropriate. However, while the sample size of 181 participants is mentioned, it would be helpful to discuss how this relates to the number of parameters in your structural equation modeling model. Structural equation modeling typically requires 5–10 participants per parameter, so a brief explanation of how the sample size meets these recommendations in relation to your model's complexity would enhance the methodological transparency.

• Qualitative content analysis:

The description of the qualitative content analysis is somewhat incomplete. While I understand that an inductive approach was used and quantification of the responses was applied, more detail on the coding process and how validity was ensured would strengthen the methodology.

• Ethics:

The ethical considerations, such as informed consent and data protection, are addressed, but it would be useful to clarify whether the study received ethics approval.

Results:

The results clearly address the research questions and objectives, providing valuable insights into primary care professionals' knowledge, attitudes, and behaviors regarding physical activity promotion. In the results section, you state:

"To improve the model fit, a recommended procedure is to remove some variables and or include additional paths, or inclusion of loops (covariance) that correlates the observed variables together."

While this is a valid methodological consideration, it would be helpful to specify who recommends this or link it explicitly to the references in your methods section. This would enhance clarity and ensure that readers understand the basis for these statistical decisions.

Discussion:

While the discussion effectively continues from the results, it lacks depth and reflection to fully contextualize the findings. I encourage the authors to provide a more analytical and interpretative discussion, exploring how the results contribute to the broader understanding of physical activity promotion in primary care and what they imply for future practice and research.

• The discussion could be improved by more explicitly tying the findings back to the introduction and the issues raised there. Reflecting on how the findings address the knowledge gaps or objectives stated in the introduction would strengthen the overall structure.

• The manuscript identifies barriers such as time constraints, limited knowledge of physical activity guidelines, and the preference for supervised programs, but it lacks concrete recommendations for addressing these issues. Providing practical strategies, such as organizational changes or targeted interventions, would enhance the practical relevance of the findings.

• The manuscript touches on important themes, such as the role of supervised programs and new staff roles like first contact physiotherapists and link workers. However, the novelty of these findings could be articulated more clearly. For example, how do these insights build on or challenge previous research? Highlighting the unique contributions of the study, particularly in relation to these professional roles, would make its value to the literature and practice more evident.

• The manuscript discusses the confidence of healthcare professionals in providing physical activity advice but raises questions about the quality and frequency of physical activity promotion in practice. Expanding on the possible reasons behind this disconnect—such as organizational barriers, knowledge gaps, or resource constraints—would add depth to the discussion.

Conclusions:

The conclusions summarize the study's findings effectively, but while they highlight barriers to physical activity promotion, they could include practical recommendations or next steps to address these issues. For example, strategies for increasing knowledge of physical activity guidelines or encouraging lifestyle-based physical activity, such as walking, could be discussed in greater detail. Additionally, the inclusion of newer roles like first contact physiotherapists and link workers provides an opportunity to explore how these positions might influence the landscape of physical activity promotion. Highlighting this potential impact would further underscore the relevance and novelty of the study.

VERSION 1 - AUTHOR RESPONSE

Reviewer's comment	Response and actions
I recommend removing all, or at least most, of the abbreviations and using the full terms for clarity.	We appreciate the reviewer for noting the excessive use of abbreviations throughout the manuscript. We agree that overuse of abbreviations can disrupt the flow and readability of the text.<br>In response, we have removed all abbreviations except for physical activity (PA), general practitioner (GP) which are commonly used and central to the focus of our study.
Additionally, I suggest mentioning the sampling strategy (convenience sampling) as a limitation, as it may affect the representativeness and generalizability of the study's findings.	We thank the reviewer for this thoughtful suggestion. We agree that it is important to acknowledge that the use of convenience sampling may affect the representativeness and generalisability of the study findings.

	In response, we have revised the limitations section of the manuscript to explicitly reflect this point. The updated statement now reads:<br><br>"The relatively small sample size, and the use of convenience sampling strategy may have affected the representation and generalisability of findings" (Pg 2. Line 30; Pg 10. Line 244-246).
Sample size and nonresponse analysis:<br>The manuscript does not report on the initial sample size or how many participants were approached but declined to participate. It would be beneficial to include information on the nonresponse rate and consider a nonresponse analysis, where you compare the demographic characteristics of respondents and nonrespondents. This would help assess selection bias and provide insight into how representative the sample	We thank the reviewer for highlighting this important point regarding sample size and the lack of non-response analysis. We agree that clarifying this aspect is essential for enhancing the rigour and transparency of the study.<br><br>We have updated the manuscript to reflect this justification and to provide greater clarity on the sampling rationale. The updated statement reads:<br>"Though, a convenient nonprobability sample lacks a predetermined or statistically calculated sample size, it has been shown to be methodologically acceptable when priority is accessibility and feasibility over representativeness, especially in research settings where the target population is difficult to access, such as healthcare professionals. Furthermore, this sampling strategy was deemed appropriate, given that the focus was on generating hypotheses rather than on generalisability of findings" (Pg 3. Line 70-73.)<br><br>Regarding the **non-response rate,** we acknowledge the importance of reporting this metric. However, due to the nature of our recruitment strategy (snowball sampling and distributed the survey via social media platforms and internal mailing lists of professional organizations) it was not possible to determine the total number of individuals who received or viewed the survey invitation. This limitation prevented us from calculating an accurate response rate. Consequently, a traditional non-response rate could not be calculated.<br><br>We have updated the manuscript to reflect this justification and to provide greater clarity on the sampling rationale. The updated statement reads:<br><br>"Due to the use of snowball sampling and survey distribution via social media and internal mailing system of the professional groups it was not possible to determine the total number of individuals who received or viewed the survey invitation. As such, a traditional non-response rate could not be determined" (Pg.10. Line 249-251)

Survey adaptation and outcomes: While the outcomes of the study are clearly defined and aligned with the research objectives, there is limited information about the process of adapting the survey from previous studies. To enhance transparency, I recommend providing more detail on how the survey was tailored to the context of this study and whether any validation or psychometric evaluation was performed on the adapted questions. Clarifying the adaptation process would strengthen the connection between the survey design and the well-defined outcomes, ensuring that the measures used are valid and reliable.	We thank the reviewer for the comments on survey adaptation and outcomes. We agree that more clarity is needed in this regard to enhance transparency.<br><br>In response, we have now provided further details on how the survey was developed and tailored to our specific context. We have now acknowledged that the survey items were developed from previous literature on PA promotion practices. In addition, we conducted a pilot test of the survey with four primary care professionals in England to assess its clarity and relevance.<br><br>The updated statement on survey development now reads:<br><br>"Survey items were developed following a literature review on PA promotion in primary care. Items assessing knowledge of physical activity guidelines, as well as attitudes and behaviours related to physical activity promotion, were adapted from a study examining the knowledge, attitude and PA promotion practices among primary care providers and oncologists. Items assessing confidence in giving PA advice were drawn from a study on GPs' PA promotion practices [9]. Items on roles and practices were adapted from a review of primary care behavioural counselling interventions while those on barriers were based on a systematic review of PA promotion delivery in primary care. (Pg 4. Line 84-88)<br><br>The updated statement on the survey pilot reads:<br><br>"In addition, to assess the clarity of the questions, pilot testing of the survey was conducted amongst GPs (n=3), practice nurses (n=2), first contact physiotherapists (n=1), and link workers (n=1). Feedback from the pilot testing indicated that the survey was generally well understood, with only minor syntactic modifications required to improve clarity and flow. For example, a question on PA promotion practice originally phrased as "I signpost patients to physical activity services" was revised to " I refer patients to a local exercise/PA programme?" to ensure broader understanding" (Pg. 4. Line 94-97)<br><br>Furthermore, we have acknowledged that the absence of validated questionnaires on this specific topic led to a pragmatic decision to develop survey items from previous surveys. We have made sure to note this limitation in the revised manuscript, highlighting that the adaptation was

	based on available resources and prior work in the field. We have now provided an updated statement, and it reads:<br><br>“Another limitation of this study is the absence of validated questionnaires on this topic. As a result, pragmatic decisions were made regarding the questions used, which were developed from previous research in the field.” (Pg 10. Line 251-252)
Statistical analysis:<br>The detailed explanation of the statistical methods used, particularly the use of structural equation modelling, bootstrapping, and the fit indices (standardized root mean square residual and comparative fit index), is appropriate. However, while the sample size of 181 participants is mentioned, it would be helpful to discuss how this relates to the number of parameters in your structural equation modelling model. Structural equation modelling typically requires 5–10 participants per parameter, so a brief explanation of how the sample size meets these recommendations in relation to your model's complexity would enhance the methodological transparency.	We thank the reviewers for highlighting the need to provide further justification for the sample size of 181, particularly in relation to the number of parameters included in the analysis.<br><br>In response, we have now clearly justified the sample size of 181 in relation to the parameters in our hypothetical model. Specifically, our model includes 15 parameters, and we have confirmed that the sample size exceeds the commonly recommended 10:1 participant-to-parameter ratio, which is generally considered sufficient for ensuring reliable statistical results. We have updated the manuscript to reflect this additional information, and the revised statement now reads:<br>“The hypothetical model included four predictor observed variables: PA knowledge, PA attitude, PA levels, and confidence to provide PA advice as predictors of the outcome variable (PA promotion). The path analysis model estimated approximately 15 parameters (see Figure 1) namely four regression paths (i.e., from each predictor → PA Promotion), four variances (i.e., one for each predictor), six covariances among predictors and one error variance for the outcome variable. With a sample size of 181 participants and 15 parameters, the study exceeds the commonly recommended 10:1 participant-to-parameter ratio [29] and meets established guidelines for sample adequacy in path analysis using observed variables [29]. Therefore, the sample size is considered sufficient to yield stable and interpretable model estimates” (Pg. 5. Line 113-118)
Qualitative content analysis:<br>The description of the qualitative content analysis is somewhat incomplete. While I understand that an inductive approach was used and quantification of the responses was applied, more detail on the coding process and how validity was ensured would strengthen the methodology.	We thank the reviewers for highlighting the need to provide further information on the content analysis. We agree that additional details are necessary to ensure the credibility of the analysis.<br>In response, we have now clarified the coding process of the content analysis. Specifically, we have added that the first author was involved in the initial coding and theme generation. Additionally, another author with more experience in qualitative research reviewed the codes and themes, offering feedback and suggesting refinements to strengthen the analysis. The updated statement now reads:

	"The first author (JO) conducted the initial coding by carefully reading and re-reading the responses, generating preliminary codes grounded in the data. These codes were then grouped into broader categories, from which initial themes were developed. To broaden interpretations and establish credibility, a second author, (SH), with a wealth of qualitative researcher experience, reviewed the codes, themes and offered feedback and suggested refinements. This collaborative and iterative process strengthened the analysis and supported the development of coherent themes grounded in the data" (Pg 5. Line 105-109).
In the results section, you state:<br>"To improve the model fit, a recommended procedure is to remove some variables and or include additional paths, or inclusion of loops (covariance) that correlates the observed variables together."<br>While this is a valid methodological consideration, it would be helpful to specify who recommends this or link it explicitly to the references in your methods section. This would enhance clarity and ensure that readers understand the basis for these statistical decisions.	We thank the reviewer for this insightful comment. We agree that clarifying the source of the recommended model modification procedures would enhance the methodological transparency and strengthen the rationale behind our statistical decisions.<br><br>In response, we have revised the statement in the results section to explicitly reference established methodological guidance. Specifically, we have now cited Kline, 2023 (Principles and practice of structural equation modelling. Guilford publications: 2023 May 24), a source commonly used in structural equation modelling (SEM) literature (119,358 citations) to support the recommended practice of improving model fit. The updated statement now reads:<br><br>"To improve the model fit, a recommended procedure is to remove some variables and or include additional paths, or inclusion of loops (covariance) that correlates the observed variables together [28]" (Pg 7. Line 159-160).
The discussion lacks depth and reflection to fully contextualize the findings. I encourage the authors to provide a more analytical and interpretative discussion, exploring how the results contribute to the broader understanding of physical activity promotion in primary care and what they imply for future practice and research	We thank the reviewer for this valuable feedback. We agree that a more analytical and interpretative discussion would strengthen the manuscript by better contextualising the findings within the broader literature on physical activity (PA) promotion in primary care. In response, we have revised the discussion section to provide deeper reflection on the significance of our findings.<br><br>Similar to previous studies, we highlight that while many primary care professionals report confidently giving PA advice. However, few could cite the guidelines, suggesting their advice may lack specificity or alignment with evidence-based recommendations. We noted that our findings extend this issue to link workers, highlighting the need for consistent guideline training across roles. We highlighted the importance of communicating both formal WHO PA guidelines and the

	broader "move more" message, particularly given common patient-reported barriers. Ultimately, we suggest that consistent PA promotion depends not only on knowledge but also on healthcare providers' confidence and perceptions of patients' capacity to engage in recommended levels of activity (Pg 8. Line 184-193)<br><br>Consistent with previous research, we found that healthcare providers seldom engaged in key PA promotion practices, such as follow-up and behaviour change support, largely due to time constraints, limited training, and perceived patient disinterest. However, first contact physiotherapists and link workers reported more engagement in these areas, likely due to longer appointments and a scope of practiced focused on PA. While these roles enhance PA promotion capacity, their limited patient access raises concerns about reach and equity. We emphasized the GP's pivotal role in reaching diverse populations and advocated for a more integrated, team-based approach to PA promotion in primary care (Pg 9. Line 203-215)<br><br>We observed that, consistent with previous research, most primary care professionals do not routinely assess PA likely due to time constraints. However, first contact physiotherapists were more likely to do so, possibly reflecting the centrality of PA in their role or greater flexibility in consultations. It remains unclear whether these assessments use formal tools or rely on clinical judgement. We interpret this as a potential opportunity for physiotherapists to enhance PA assessment in routine primary care. We also noted that further research is needed to understand current assessment practices, underlying rationales, and patient views on meaningful PA assessment (Pg 8. Line 194-202)<br><br>In summary, we have clarified that fewer than half of healthcare providers were able to accurately cite the PA guidelines, and that routine PA assessment and behaviour change support were infrequently reported. As a result, we noted that the quality and specificity of the PA advice being offered remains unclear. While we acknowledge that improving provider knowledge is important, we argue that this alone is unlikely to lead to meaningful changes in PA promotion practices. Accordingly, we have now emphasized the need for further research to better understand the nature of PA advice currently being provided, the characteristics of the patients who receive it, and the underlying rationale informing such practices (Pg 11.Line 270-278).

The manuscript identifies barriers such as time constraints, limited knowledge of physical activity guidelines, and the preference for supervised programs, but it lacks concrete recommendations for addressing these issues. Providing practical strategies, such as organizational changes or targeted interventions, would enhance the practical relevance of the findings	**PA behavioural change support:** In response, we have noted that first contact physiotherapist and link workers compared to other health providers reported promoting PA behavioural change support. We noted that that more time, and higher confidence in behaviour changes counselling were possible reasons for more engagement in behavioural support.<br>Based on our findings we recommended the following:<br><br>• Providing motivational interview and PA behaviour change training for first contact physiotherapist and link workers (Pg 8-9. Line 212-214)<br>• While the involvement of first contact physiotherapist and link workers could boost PA promotion capacity, we noted they their limited access to patients compared to GPs raises concerns about reach and equity. To address this, we now emphasise the need for a more integrated model in which PA promotion is reconceptualised as a shared responsibility across the primary care team. We have also expanded our recommendations to include system-level interventions, such as incorporating PA-related quality indicators into performance frameworks. Additionally, we suggest embedding behaviour change competencies into GP training and revalidation and promoting interdisciplinary collaboration to help normalise PA promotion as routine care. Finally, we highlight the potential of digital tools to support motivational screening, brief interventions, and automated follow-ups, which may ease time constraints while enhancing continuity of care. We believe these revisions strengthen the manuscript by offering a more comprehensive and pragmatic strategy for embedding PA promotion in primary care. (Pg 8-9. Line 212-214)<br><br>**PA assessment:** We noted that a lack of routine PA assessment. We have provided the following recommendations that could enhance PA assessment. The recommendations provided are as follows (Pg 1. Line 257-259):<br>1. Targeted training in PA assessment in primary care

	2. Incentivising routine PA assessment through incorporating PA assessment into the Quality and Outcomes Framework (QOF).<br><br>Limited knowledge: We have now provided recommendations on how to overcome the challenge of limited knowledge. In the revised manuscript, we noted the following recommendations:<br>1. While there has been significant advocacy for improving GPs' knowledge in this area, we argue that similar efforts must be extended to other primary care health providers, including nurses, First Contact Physiotherapists, and social prescribing link workers (Pg 8; Line 188)<br>2. We now emphasise that while the WHO recommends at least 150 minutes of moderate-to-vigorous physical activity per week, it also highlights that any movement is beneficial to health. Accordingly, we note that clinicians should communicate both the formal guidelines and the broader “move more” message. In light of common patient-reported barriers such as time constraints and family commitments, we argue that encouraging any increase in activity can offer a realistic and accessible entry point. Finally, we highlight that consistent PA promotion depends not only on clinicians’ knowledge of guidelines, but also on their willingness and confidence to promote them both of which may be shaped by their perceptions of a patient’s capacity to engage in activity at the recommended intensity. (Pg 8; Line 189-193)<br><br>Preference for supervised PA programme: In the revised manuscript, we have clearly acknowledged an apparent mindset amongst participants favouring facility-based or supervised PA as revealed through the qualitative responses (i.e., the citing of limited availability and affordability of local PA opportunities as a barrier to PA promotion), potentially narrowing the scope of PA promotion. We have provided the following recommendations to support promotion of lifestyle activities such as walking:<br>1. GPs should prioritise independent self-managed lifestyle activities such as walking as a key form of PA. This can be supported by referrals to link workers who can connect patients who may need an extra motivation to engage in lifestyle activities such as walking (Pg 9. Line 221-223)

	2. We also recommended that as link workers become further integrated into primary care, raising awareness among clinicians of their role in facilitating lifestyle forms of PA, such as walking, will be essential. (Pg 9. Line 223-224)
The manuscript touches on important themes, such as the role of supervised programs and new staff roles like first contact physiotherapists and link workers. However, the novelty of these findings could be articulated more clearly. For example, how do these insights build on or challenge previous research? Highlighting the unique contributions of the study, particularly in relation to these professional roles, would make its value to the literature and practice more evident	We sincerely thank the reviewer for this thoughtful comment. We agree that more clearly articulating how link workers and first contact physiotherapist contribute to PA promotion is essential for demonstrating its contribution to the literature. In response, we have strengthened the discussion section to explicitly highlight the unique insights our study offers. While much of the existing literature has concentrated on the role of general practitioners in PA promotion, our study broadens this perspective by examining the attitudes, practices, and challenges across a wider range of emerging roles within the primary care workforce particularly First Contact Physiotherapists and Social Prescribing Link Workers. Below are specific information on how findings from our research build on previous findings:<br><br>Knowledge of PA guidelines: We clarify that, consistent with previous research, most providers were unable to cite current PA guidelines, raising concerns about whether their advice aligns with evidence-based recommendations. While prior studies have identified this gap among GPs, practice nurses, and physiotherapists, our findings extend this issue to link workers. We have therefore emphasised the need to embed PA guideline training across all professional groups as part of both induction and ongoing professional development (Line 8. Line 186-188).<br><br>PA assessment: Consistent with previous research, most primary care professionals did not routinely assess PA. We noted that time constraints in previous studies have been reported as a challenge to PA assessment. We however highlighted that more first contact physiotherapists reported routinely assessing PA, which may reflect the central role of PA in their clinical practice or possibly greater time flexibility within their consultations. We noted that it remains unclear whether these assessments are based on validated tools or informal judgement. These findings suggest that first contact physiotherapists may play a key role in embedding PA assessment into routine care. We have also highlighted the need for further research to understand how PA is currently assessed across professions, the rationale behind these practices, and patients’ views on acceptable and meaningful forms of PA assessment (Pg. Line 194-.202).

	**PA behaviour change practice**: Similar to previous studies, we noted that PA promotion components related to PA behaviour change is often not promoted by GPs possibly because of time, perceived patient disinterest and a lack of confidence to support PA behaviour change. Findings from this study showed that first contact physiotherapists and Link workers reported promoting PA components related to PA behaviour change support. We therefore expanded the discussion to emphasise that first contact physiotherapists and Link workers have the potential to enhance the capacity of primary care to promote PA, particularly given GPs' limited time and clinical demands. (Pg 9. Line 203-215)
The manuscript discusses the confidence of healthcare professionals in providing physical activity advice but raises questions about the quality and frequency of physical activity promotion in practice. Expanding on the possible reasons behind this disconnect—such as organizational barriers, knowledge gaps, or resource constraints—would add depth to the discussion	We thank the reviewer for highlighting the importance of exploring the reasons behind the disconnect between healthcare professionals' confidence in providing PA advice and the actual frequency and quality of PA promotion in practice.<br>• PA guidelines: In response, we have expanded the discussion section to address the potential barriers contributing to this gap. While healthcare professionals, often express confidence in providing PA advice, several key factors likely hinder the promotion of recommended PA guidelines. We noted that asides from a lack of knowledge of the knowledge of PA guidelines, the healthcare providers willingness and confidence to promote them may also depend on their perception of a patient's capacity to engage in activity at the recommended intensities. We therefore recommended studies exploring the relevance and the willingness of health providers to promote the PA guidelines. (Pg 8. Line 190-193)<br>• PA behaviour change promotion: We have now noted that a possible reason why few GPs and practice nurses reported promoting PA behavioural change support could be as a result of a lack of time, confidence and perceived patient disinterest in PA advice. Concerning perceived patient disinterest in PA advice, we noted that this may be related to communication misperception rather than genuine patient disengagement, highlighting the need for more personalised and outcome-oriented communication strategies. (Pg 9-10. Line 226-235)
Conclusions:<br>The conclusions summarize the study's findings effectively, but while they highlight	We appreciate the reviewer's insightful suggestion to expand the conclusions by including practical recommendations and

barriers to physical activity promotion, they could include practical recommendations or next steps to address these issues. For example, strategies for increasing knowledge of physical activity guidelines or encouraging lifestyle-based physical activity, such as walking, could be discussed in greater detail. Additionally, the inclusion of newer roles like first contact physiotherapists and link workers provides an opportunity to explore how these positions might influence the landscape of physical activity promotion. Highlighting this potential impact would further underscore the relevance and novelty of the study.	discussing the potential impact of newer roles, such as first contact physiotherapists and link workers, on PA promotion.<br><br>In response, we have revised the conclusions section to provide more specific recommendations for addressing the identified barriers to PA promotion. These recommendations now include strategies to **increase knowledge of PA guidelines** among primary care professionals, emphasising the importance of training, Additionally, we have highlighted the need for **targeted initiatives** aimed at encouraging **lifestyle-based physical activity**, such as walking and cycling, which are both accessible and sustainable options. Moreover, we have expanded on the potential impact of **newer roles** within primary care, such as **first contact physiotherapists and Link Workers**, to enhance PA promotion within primary care. We discuss how these roles can not only **fill gaps in PA promotion** but also provide **a more holistic approach** by offering patients personalized and accessible PA options. By integrating these professionals into the broader PA promotion strategy, primary care teams can provide more comprehensive and effective support for patients looking to engage in regular PA. The updated conclusion reads:<br><br>"A high proportion of primary care professionals report regularly providing PA advice to patients and express confidence in doing so. However, since less than half can accurately cite the PA guidelines and routine PA assessment and behaviour change support are rarely reported, the quality and specificity of the PA advice remain unclear. While improving knowledge is necessary, it is unlikely to be sufficient on its own. Given the infrequent PA assessment and behaviour change support observed, further research is warranted to more fully explore the nature of PA advice provided, the patients who receive it, and the reasoning behind such PA promotion practices. Additionally, findings suggest a prevailing mindset among primary care professionals that favours facility-based or supervised PA programs, potentially narrowing the scope of PA promotion. Perceived patient disinterest is frequently cited as a barrier, though this may reflect a communication misperception rather than actual disengagement. With the potential for first-contact physiotherapists and link workers to enhance capacity for promoting PA in busy primary care settings, this study calls for stronger integration of these roles within multidisciplinary teams to foster greater patient engagement in PA.<br>" (Pg 11. Line 270-278).

VERSION 2 - REVIEW

Reviewer **1**

Name **Nordgren, Lena**

Affiliation **Uppsala Universitet, Department of Public Health and Caring Sciences**

Date **24-Jun-2025**

COI

The authors have clearly made a great effort to revise the manuscript, and the result is a significantly improved paper. I appreciate the care taken in addressing the comments and the thoughtful way the revisions were carried out. From my perspective, the manuscript is now in a publishable state and does not require further changes.
